# Compression Response of Silicone-Based Composites with Integrated Multifunctional Fillers

**DOI:** 10.3390/polym17040500

**Published:** 2025-02-14

**Authors:** Ingyu Bak, Jihyeon Kim, Andrew Jacob Ruba, David John Ross, Kwan-Soo Lee

**Affiliations:** 1 MPA-11: Materials Synthesis & Integrated Devices, Los Alamos National Laboratory, Los Alamos, NM 87545, USA; igbak@lanl.gov (I.B.); jhkim@lanl.gov (J.K.); ajruba@lanl.gov (A.J.R.); 2 MST-7: Engineered Materials, Los Alamos National Laboratory, Los Alamos, NM 87545, USA; dross@lanl.gov

**Keywords:** polydimethylsiloxane, polymer composite, filler, boron, hollow glass microballoon, tungsten

## Abstract

Polydimethylsiloxane (PDMS) is known for its exceptional mechanical properties, chemical stability, and flexibility. Recent advancements have focused on developing functional PDMS composites by integrating various functional fillers, including polymers, ceramics, and metals, for advanced applications such as electronics, medical devices, and aerospace. Consequently, there is a growing need to investigate PDMS composites to achieve higher filler loadings offering enhanced mechanical performance. This study addresses this need by utilizing the high molecular weight (MW) PDMS resin we have developed, offering its high elongation capacity of up to >6500%. We incorporated boron (B), hollow glass microballoons (HGMs), and tungsten-coated hollow glass microballoons (WHGMs) into the developed high MW PDMS. The resulting composites demonstrated excellent elastic properties and significant compression resilience (35–80%) and elastic modulus (1.28–10.15 MPa) at high filler loadings (~60 vol.%). Specifically, B/PDMS composites achieved up to 67.6 vol.% of B, HGM/PDMS composites held up to 68.6 vol.% of HGM, and WHGM/PDMS composites incorporated up to 54.0 vol.% of WHGM. These findings highlight the potential of high MW PDMS for developing high-performance PDMS composites suitable for advanced applications such as aerospace, automotive, and medical devices.

## 1. Introduction

Polydimethylsiloxane (PDMS) is an inorganic polymer widely used across various industries due to its remarkable properties, including chemical inertness, thermal stability, and flexibility [1,2,3,4,5,6]. These attributes make PDMS a favored material in fields such as aerospace, automotive, and medical devices [7,8,9]. The integration of fillers into PDMS composites has further expanded its utility, enabling significant improvements in mechanical performance and functionality [10,11]. Silica and carbon materials have been widely studied as fillers to modify the thermal, electrical, and mechanical properties of PDMS [12,13,14,15]. Additionally, diverse fillers, such as ceramics, metals, and polymers, have been explored to tailor PDMS composites for specific applications [11,16,17,18]. Building on these efforts, contemporary research focuses on integrating high-performance fillers to impart additional functionalities [19,20].

Unlike silica or carbon fillers that have been used for PDMS resin reinforcement, our selection of boron (B), hollow glass microspheres (HGMs), and tungsten-coated hollow glass microspheres (WHGMs) is driven by their ability to impart multifunctionality. B has been particularly noted for its contribution to radiation shielding in polymer composites [21,22,23,24]. It enhances the stability and protective performance of PDMS composites, making it ideal for applications requiring shielding from ionizing radiation, such as aerospace and nuclear environments. HGM is commonly used to reduce the density of PDMS composites while maintaining or enhancing mechanical strength [25,26,27,28,29]. This makes HGMs suitable for applications demanding lightweight materials with robust mechanical properties. Studies have demonstrated that incorporating HGMs into PDMS significantly reduces thermal conductivity and increases elastic modulus, making these composites effective for thermal insulation and structural applications [26,29]. Additionally, HGMs improve impact resistance by providing energy-absorbing mechanisms that enhance material toughness [25,27,28,30]. WHGM increases the stiffness and wear resistance of PDMS composites [31,32,33]. WHGM also improves radiation shielding properties, which is critical for space exploration and aerospace applications [33,34,35].

This study investigates the high molecular weight (MW) PDMS resin developed by our team. It is composed of a PDMS macromer and can be polymerized to achieve an ultrahigh molecular weight. The ultrahigh molecular weight enables crosslinking through chain entanglement, providing mechanical strength and exceptional elongation capabilities exceeding 6500%. The research focuses on enhancing the mechanical properties and maximizing filler loading in PDMS composites by leveraging the resin’s unique characteristics. We examine the effects of incorporating B, HGMs, and WHGMs on the mechanical performance and physicochemical properties of PDMS-based composites. The results demonstrate the potential of high MW PDMS to accommodate high filler content while enhancing mechanical properties, making it promising for applications in flexible electronics, medical devices, and lightweight structural components.

## 2. Materials and Methods

### 2.1. Sample Preparation

The PDMS-based polymer matrix in this study refers to a composite material consisting of 70 wt.% high molecular weight polydimethylsiloxane (HMW-PDMS) and 30 wt.% dimethyldichlorosilane-treated silica (surface area: 125 m²/g, Gelest, Inc., Morrisville, PA, USA). The boron (B, 140 mesh, 2.3 g/cm^3^) and hollow glass microballoon (HGM, commercial name 3M™ Glass Bubble S32HS; mean size of 25 µm, 0.32 g/cm^3^) were purchased from 3M Company, St. Paul, MN, USA. The tungsten-coated hollow glass microballoon (WHGM, mean size of 25 µm, 0.87 g/cm^3^) was prepared using a DC sputtering system (Model VTC-16-PW, MTI Corporation, Richmond, CA, USA) with in-chamber powered agitation provided by a vibration motor mounted to the powder reservoir. For each powder coating run, 2.6 g of HGM S32HS were loaded into the powder reservoir, and a 2” diameter circular tungsten target was installed in the sputter source. The system was allowed to pump to its base pressure of 6.5 × 10^−5^ Torr followed by the introduction of 4 sccm of 99.9999% argon to achieve a chamber pressure of 10 mTorr. The DC power supply was ramped to 50 W, with a 5 min burn-in period before initiating coating. Powder agitation was maintained during the 12.5 h coating process.

The fillers, B, HGMs, and WHGMs were homogeneously blended into the in-house PDMS-based polymer matrix using a planetary mixer (Thinky ARV-310, THINKY USA Inc., Laguna Hills, CA, USA) with a cooling adapter at 2000 rpm for 2 min. In this study, the mixture of the PDMS-based polymer matrix and the fillers is referred to as PDMS composites. The filler content was systematically increased in increments to evaluate the maximum feasible loading while maintaining processability and mechanical stability. After mixing, the samples were poured into molds and compressed using a heated press (Model M 3853, Carver, Inc., Wabash, IN, USA) at a pressure of 0.5 tons for 30 min. Following this compression, the samples were thermally cured in their molds using a BINDER™ heating oven with forced convection (BINDER™ FD 115, BINDER GmbH, Tuttlingen, Germany) at 130 °C for 8 h. Once cured, the samples were detached from the molds, and their properties were characterized. Table 1 provides a detailed summary of the physical properties of the PDMS composites, including composition, hardness (Shore A), density, and void content.

### 2.2. Optical Microscopy

Cross-sectional images and surface roughness of the samples were captured using a onfocal microscope (VHX-6000, Keyence Corporation of America, Itasca, IL, USA). Strand dimensions were measured using Keyence analysis software (Version 1.4.25.19). Surface roughness was measured using the microscope’s real-time depth composition function for 3D image mapping.

### 2.3. Thermogravimetric Analysis (TGA)

A TA Instruments Discovery Series 550 (New Castle, DE, USA) was used to perform thermogravimetric analysis (TGA) on samples weighing between 5 and 10 mg. The analysis involved equilibrating the samples at 50 °C, followed by heating them to 900 °C at a rate of 10 °C/min under a nitrogen atmosphere. The degradation temperature, defined as the temperature at which a 5% mass loss occurred, was recorded. Peak degradation temperatures were identified using the first derivative of the weight loss curve.

### 2.4. Differential Scanning Calorimetry (DSC)

Differential scanning calorimetry (DSC) measurements were conducted using a TA Instruments Discovery Series 2500. Approximately 5 to 10 mg of the sample was subjected to the following thermal cycle: heating from −150 °C to 100 °C at a rate of 10 °C/min (first heating ramp), holding isothermally for 5 min, cooling from 100 °C to −150 °C at a rate of 10 °C/min, holding isothermally for another 5 min, and then reheating from −150 °C to 100 °C at 10 °C/min (second heating ramp). The second heating ramp data were used for analysis. Key parameters such as the glass transition temperature (*T_g_*), melting temperature (*T_m_*), crystalline temperature (*T_c_*), enthalpy of melting (Δ*H_f_*), and enthalpy of crystallization (Δ*H_c_*) were determined using TRIOS software (Version 5.7.2.101). Crystallinity (*X_c_*) was calculated using the formula below, where 61.19 J/g represents the theoretical enthalpy of fusion for a fully crystalline PDMS polymer [34,36] as follows:

$$X_{c}\left(\%\right)=\left(\frac{{∆H}_{f}}{61.19}\right)\times 100$$

### 2.5. Mechanical Testing

The mechanical properties of circular sample pads, each with a diameter of 4.5 cm, were assessed using an Instron-3343 tabletop uniaxial material testing machine equipped with a 1 kN load cell from Instron Norwood USA. The compression tests were conducted in accordance with ASTM D575 [37], which specifies the standard test methods for rubber properties in compression. The pads were compressed at a steady rate of 20 mm/min. Cyclic compression tests were performed to evaluate the viscoelastic properties and energy dissipation of the composites. These tests involved subjecting the samples to cyclic loading and unloading at different pressures, specifically 0.1, 0.2, and 0.6 MPa, with each cycle repeated four times. To minimize Mullins effects, data from the 4th cycle are reported in this study. The elastic modulus was calculated from the linear region of the stress–strain curve obtained during compression testing. Hardness tests were carried out using a benchtop HPE II Zwick Roell Shore hardness tester, adhering to the ASTM D2240 [38] standard. This procedure requires samples to have a minimum thickness of 6 mm to minimize measurement errors. Each sample was tested at five locations, with at least three trials performed for each formulation.

### 2.6. Density Determination

Density measurements were conducted using gas pycnometry (Ultrapyc 5000, Anton Paar GmbH, Graz, Austria). Helium was chosen as the investigation gas because of its ideal gas behavior and ability to penetrate pores approximately 2.6 Å and larger [39,40]. In this study, all density measurements are averaged across five measurements. The void content, or void volume fraction, was determined using density measurements using ASTM D2734 [41,42]. The void content (*V_v_*) is calculated from the relative difference between the theoretical density ($\rho_{T}$) and the measured density ($\rho_{M}$) of the composite material. The formula for void content is given by the following:

$$V_{v}=100-\rho_{M}\left(\frac{W_{p}}{\rho_{p}}+\frac{W_{f}}{\rho_{f}}\right)$$

where *W* and *ρ* denote the weight percentage and density and subscripts *p* and *f* refer to the PDMS-based polymer matrix and filler, respectively.

## 3. Results

### 3.1. Characterization of the PDMS-Based Polymer Matrix Properties

**Figure 1:**
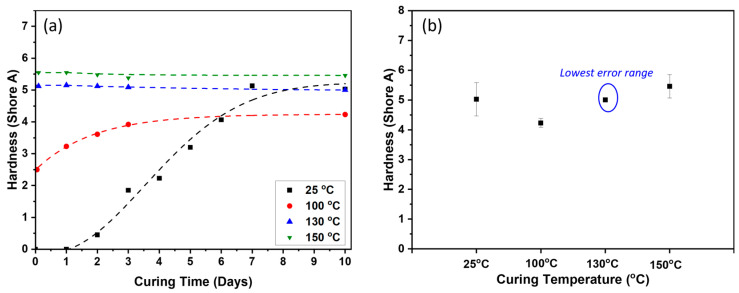
The hardness of the prepared PDMS-based polymer matrix for each curing condition to determine the optimized curing time. (**a**) The different curing times followed by different curing temperatures and (**b**) the error range of each cured sample.

When the PDMS-based polymer is cured, it has excellent mechanical performance, with an elongation exceeding 6500% at a tensile testing rate of 100 mm/min. The PDMS-based polymer matrix was prepared by incorporating 30 wt.% of dimethyldichlorosilane-treated silica to enhance the matrix’s mechanical properties. To solidify this polymer matrix formulation, a high-temperature catalyst has been used for the hydrosilylation reaction [6,43]. Various curing conditions have been tested to understand the curing behaviors with different temperatures at 25, 100, 130, and 150 °C. Figure 1a illustrates that curing at 25 °C requires more than 7 days to complete, leading to significant variability in hardness. In contrast, curing at 100 °C takes over 3 days and results in a relatively low hardness of 4 Shore A. When the curing temperature is increased to 130 °C and 150 °C, the hardness improves to values above 5 Shore A, with curing times of 8 h. Figure 1b shows that samples cured at 130 °C achieve the desired hardness and exhibit minimized error ranges, ensuring reproducibility in subsequent experiments. Consequently, all further curing processes were conducted at 130 °C for 8 h.

**Figure 2:**
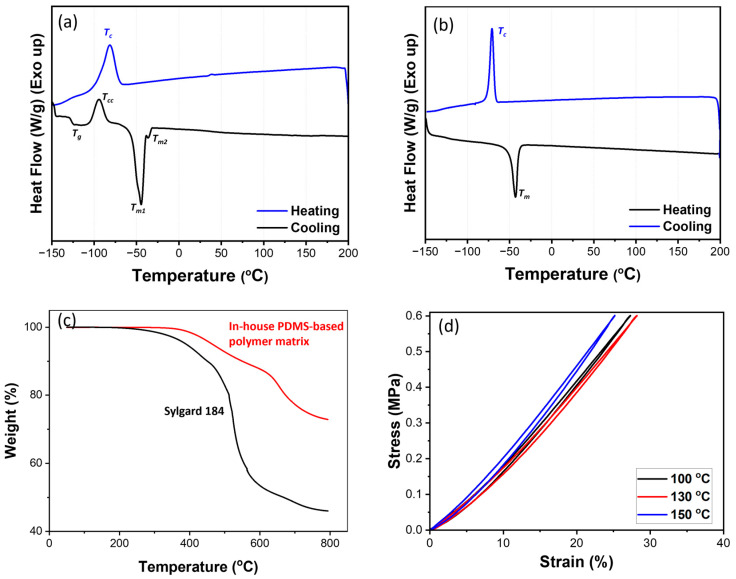
The properties of the PDMS-based polymer matrix. (**a**) DSC of the uncured PDMS-based polymer matrix, (**b**) DSC of the cured PDMS-based polymer matrix, (**c**) TGA of the cured PDMS-based polymer matrix and Sylgard 184, and (**d**) cyclic compression behavior of the PDMS-based polymer matrix under different curing temperatures.

The thermal and mechanical properties of the PDMS-based polymer matrix are shown in Figure 2. Figure 2a presents the DSC results for the uncured polymer matrix. The data correspond to uncured PDMS, as indicated by the presence of clear thermal events such as glass transition temperature (T_g_) at −127 °C, cold crystallization at −95 °C, and melting (T_m_) at −45 °C during the first heating cycle. During the second cooling cycle, the crystallization temperature (T_c_) is observed at −82 °C. The cured PDMS-based matrix exhibits a T_c_ at −82 °C and a melting temperature (T_m_) at −45 °C (Figure 2b, Figure S2). No heat emission in the higher temperature range (100–150 °C) indicates that further curing reactions have occurred. The thermal stability of the developed polymer matrix is compared with that of Sylgard 184. The cured polymer matrix exhibits superior thermal stability due to its ultrahigh MW (Figure 2c). It has a higher onset temperature and leaves more residue at 800 °C compared to Sylgard 184. The higher residue and onset temperature indicate that the in-house developed PDMS-based polymer matrix possesses a stable thermal profile, making it suitable for applications requiring high-temperature resistance. The compressive test was conducted on the polymer matrix samples cured at three different temperatures: 100 °C, 130 °C, and 150 °C (Figure 2d). The results illustrate the effect of curing temperature on the mechanical properties of the matrix. Samples cured at 150 °C exhibit higher compressive stiffness, while those cured at 100 and 130 °C display lower stiffness.

### 3.2. Characterization of PDMS Composites

**Figure 3:**
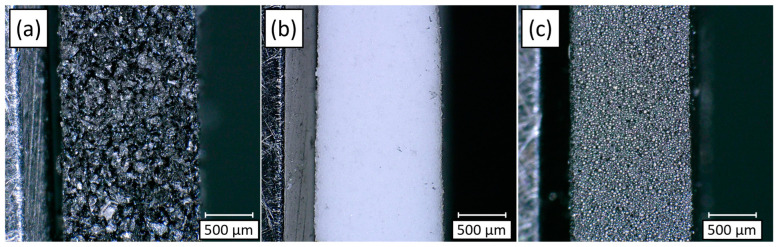
Cross-sections of PDMS composites containing (**a**) B 63.0 vol.%, (**b**) HGM 68.6 vol.%, and (**c**) WHGM 54.0 vol.% measured using a Keyence VHX-6000 microscope at 100× magnification.

The mechanical properties of PDMS composites were evaluated by examining the influence of different fillers and their respective loading contents. Figure 3 and Figures S7–S10 present cross-sectional microscopic images of PDMS composites containing various fillers, highlighting their dispersion within the polymer matrix. The cross-sectional images reveal that the fillers are uniformly distributed within the PDMS matrix, with no significant defects observed, even at high filler loadings. These images indicate that the PDMS-based polymer matrix effectively accommodates high filler content while maintaining structural integrity, suggesting strong filler–matrix interactions and good compatibility. The surface of the composite becomes rougher with filler content (Figure S1). The B/PDMS composite exhibits the roughest surface due to the amorphous nature and large size (100 µm) of boron, while HGM and WHGM contribute less to surface roughness due to their smaller size (25 µm) and round shape. Table 1 shows that the hardness of the composites increases with higher filler loadings, which is consistent across all types of fillers. The density measurements align closely with the theoretical values calculated based on the filler weight percentages. The B/PDMS shows the highest void content, ranging from 0.00 to 6.23%, which might be attributed to the amorphous shape and large mesh of the B powder. The WHGM/PDMS exhibits a void content of 0.00 to 3.77%, while the HGM/PDMS has the lowest void content, ranging from 0.00 to 2.92%. The low void content indicates a considerable filler-matrix interaction.

**Figure 4:**
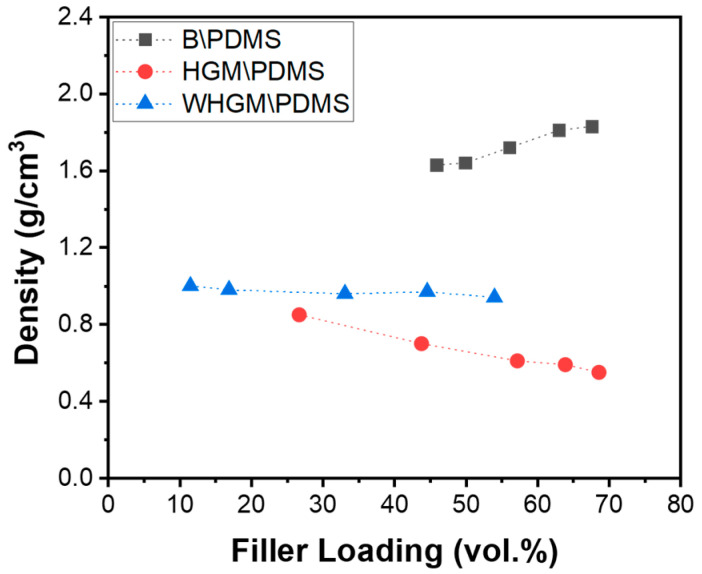
Density measurements of B/PDMS (boron, 65–85 wt.%), HGM/PDMS (HGM, 10–40 wt.%), and WHGM/PDMS (WHGM, 10–50 wt.%). The data illustrate the influence of filler type and content on the overall composite density.

Figure 4 presents the density measurements of PDMS composites with varying volume percentages of B, HGMs, and WHGMs. The densities were measured to understand how the inclusion and loading of different fillers impact the overall density of the composites. The data show a clear trend where the density of the composites is directly influenced by the type and amount of filler used. The experimental results reveal that as the B content increases from 65 to 85 wt.%, the density of the composites increases nonlinearly from 1.63 g/cm^3^ to 1.83 g/cm^3^. This nonlinear increase is attributed to the void content in the composite. The HGM-filled PDMS composites exhibit a linear density reduction due to their low density of 0.32 g/cm^3^. As the HGM content increases from 10 to 40 wt.%, the overall density of the composites decreases from 0.85 g/cm^3^ to 0.55 g/cm^3^. The density of the WHGM is nearly similar to that of the polymer matrix, resulting in a slight density reduction for WHGM/PDMS from 1.00 g/cm^3^ to 0.94 g/cm^3^, as the filler content increases from 10 to 50 wt.% (Table 1).

**Figure 5:**
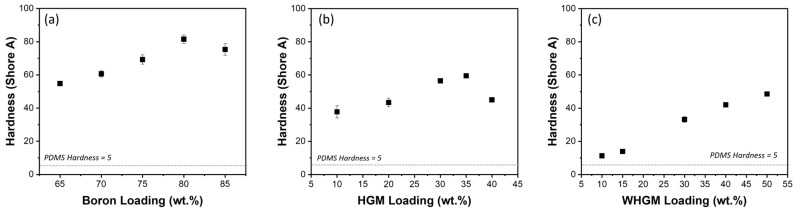
Hardness (Shore A) of PDMS composites with different weight percentages of fillers: (**a**) boron (65–85 wt.%), (**b**) HGMs (10–40 wt.%), and (**c**) WHGMs (10–50 wt.%). Error bars represent the standard deviation from five measurements.

Figure 5 presents the hardness test results of PDMS composites with varying filler content. The results indicate that the hardness of the PDMS composites is significantly influenced by the type and loading content of the fillers. The pure PDMS-based polymer matrix exhibits a baseline hardness of 5 Shore A with a density of 1.05 g/cm^3^. When boron fillers are incorporated into the PDMS-based polymer matrix, a substantial increase in hardness is observed. For example, at 65 wt.% boron, the hardness reaches 54.8 Shore A, increasing further to 81.5 Shore A at 80 wt.% boron. However, the hardness slightly decreases to 75.3 Shore A at 85 wt.%, which may be due to potential cracking caused by incomplete interaction between the metal particles and the polymer matrix. HGMs also affect the hardness of the composites. At 10 wt.% HGM, the hardness is 37.8 Shore A, which increases to 59.9 Shore A at 35 wt.%. However, at 40 wt.% HGM, the hardness decreases to 45.0 Shore A, indicating a potential limit to the reinforcing effect of HGMs at higher loadings. For WHGMs, the hardness consistently increases with loading up to 50 wt.%. At 10 wt.% WHGM, the hardness is 11.3 Shore A, increasing to 48.5 Shore A at 50 wt.%. This suggests a reinforcing effect of WHGMs on the PDMS-based polymer matrix.

### 3.3. Compressive Test Results of B/PDMS Composites

**Figure 6:**
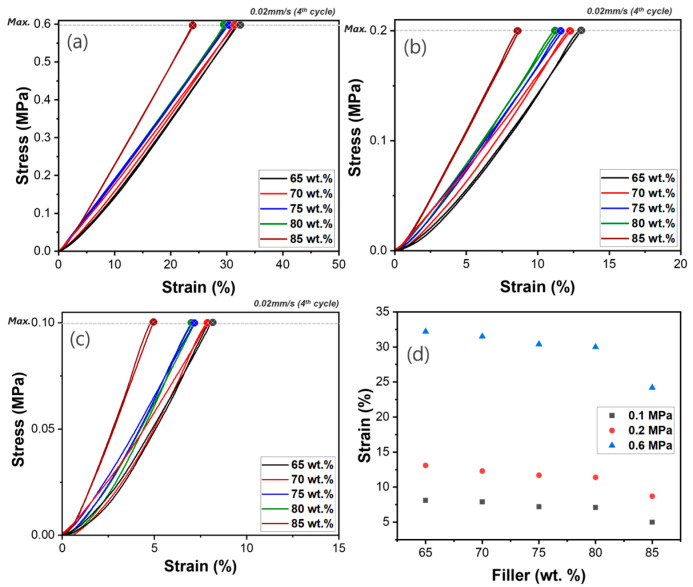
Cyclic compressive behavior of B/PDMS composites with varying B content: 65, 70, 75, 80, and 85 wt.% under different maximum stresses; (**a**) 0.6 MPa, (**b**) 0.2 MPa, (**c**) 0.1 MPa, (**d**) compressive strain as a function of the B loading amount.

**Figure 7:**
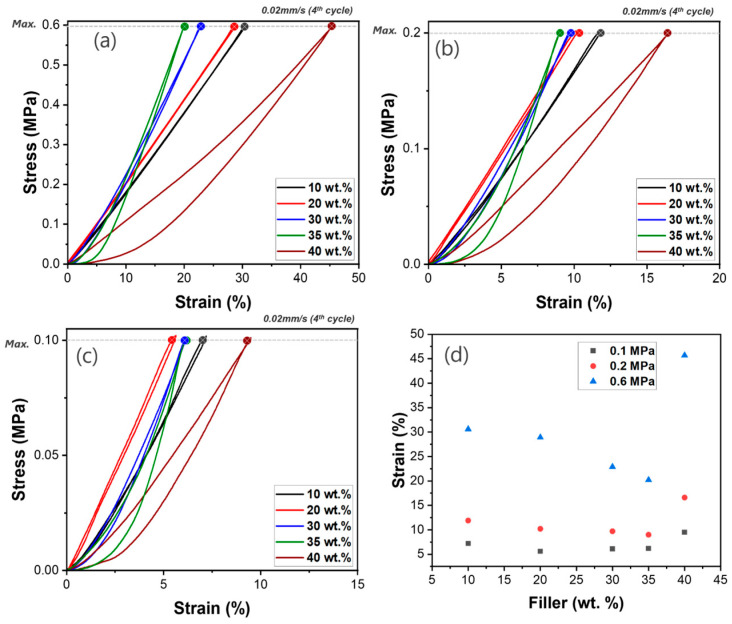
Cyclic compressive behavior of HGM/PDMS composites with varying HGM content: 10, 20, 30, 35, and 40 wt.% under different maximum stresses; (**a**) 0.6 MPa, (**b**) 0.2 MPa, (**c**) 0.1 MPa, (**d**) compressive strain as a function of the HGM loading amount.

**Figure 8:**
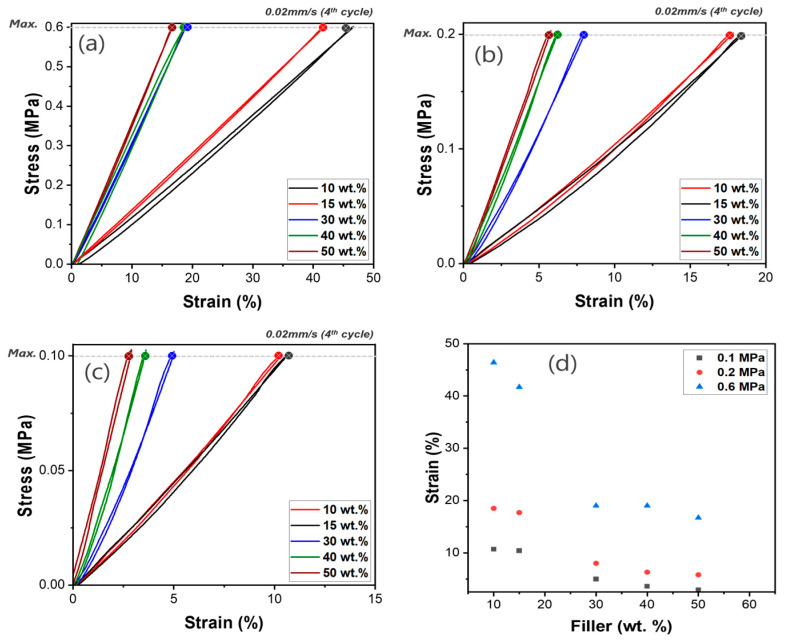
Cyclic compressive behavior of WHGM/PDMS composites with varying WHGM content: 10, 15, 30, 40, and 50 wt.% under different maximum stresses; (**a**) 0.6 MPa, (**b**) 0.2 MPa, (**c**) 0.1 MPa, (**d**) compressive strain as a function of the WHGM loading amount.

**Figure 9:**
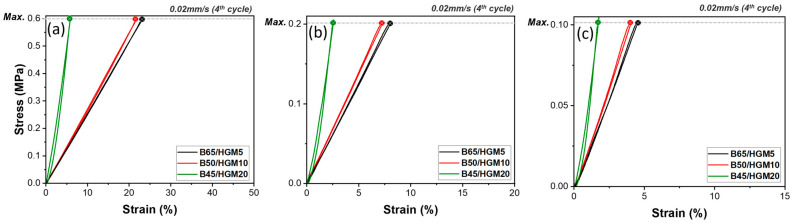
Cyclic compressive behavior of B/HGM/PDMS composites with varying B/HGM content: 45/20, 50/10, and 65/5 wt.% under different maximum stresses; (**a**) 0.6 MPa, (**b**) 0.2 MPa, (**c**) 0.1 MPa.

The mechanical properties of PDMS composites were evaluated through compressive stress testing to assess the influence of B, HGM, and WHGM fillers, as well as their respective loading contents. Figure 6, Figure 7, Figure 8 and Figure 9 provide the fourth cycle of comprehensive data on the compressive behavior of the composites under three different pressures of 0.1, 0.2, and 0.6 MPa, while Figures S3–S6 offer the full cycles for each cyclic compression test. The PDMS/B composite was not processible beyond 85 wt.%, so we investigated mechanical strength and processability in the range of 65 wt.% to 85 wt.%. The compressive stress–strain curves for PDMS composites with varying B content reveal distinct compressive behaviors (Figure 6). PDMS composites containing 65 wt.% B exhibit a compression strain of 32.2% at 0.6 MPa, as shown in Figure 6a. With an increase in B content to 70, 75, and 80 wt.%, the B/PDMS became slightly rigid, and the strain is 30.0% at 80 wt.%. As shown in Figure 6d, it is concluded that the B/PDMS composites reach a percolation limit at about 80 wt.% B, with the strain significantly reduced to 24.2% at 85 wt.% B. Similar compressive stress–strain behavior under maximum stresses of 0.1 and 0.2 MPa are displayed in Figure 6b,c. The variation in compression behavior with different B contents can be attributed to the enlarged interface between the filler–matrix interface and the inherent rigidity of B particles. As the filler content increases, the B/PDMS composite becomes more resistant to deformation under compressive loads, which is reflected in the increasing elastic modulus from 1.86 MPa at 65 wt.% B to 2.48 MPa at 85 wt.% B. This behavior is typical of composite materials where the filler acts to reinforce the matrix, improving its mechanical properties [12,14].

### 3.4. Compressive Test Results of HGM/PDMS Composites

The compressive properties of HGM/PDMS composites were evaluated to understand the effect of filler content on mechanical performance. The PDMS/HGM composite was not processable above 40 wt.%, so we examined its mechanical strength and processability between 10 wt.% and 40 wt.%. Figure 7 presents the cyclic compressive behavior of HGM/PDMS composites under three different pressures of 0.1, 0.2, and 0.6 MPa. As the HGM content increases to 35 wt.%, the compressive strain decreases from 30.6% to 20.2% at 0.6 MPa (Figure 7a), demonstrating a trend towards increased stiffness and reduced deformation under applied stress. This trend is also reflected in the elastic modulus, which increases from 1.95 MPa at 10 wt.% HGM to 2.99 MPa at 35 wt.% HGM. However, at 40 wt.% HGM, the compressive strain increases, and the elastic modulus drops to 1.31 MPa, indicating a reduction in rigidity and a higher degree of compressibility. This suggests that beyond a certain filler content, the HGM contributes softness rather than rigidity, possibly due to its deformable hollow structure and the emerged interfaces between HGMs caused by the high HGM content, which are easy to slip.

The hysteresis loops observed in the stress–strain curves indicate the energy dissipation behavior of the composites during cyclic loading. At 10–30 wt.% HGM, the hysteresis loop areas remain small. However, at 35 wt.% HGM, a significant hysteresis loop appears and, at 40 wt.% HGM, the hysteresis loop area becomes larger, suggesting high energy dissipation. Compared to the B/PDMS composite, the HGM in composites experience localized stress concentrations and microstructural damage during compression, leading to higher energy dissipation and larger hysteresis loop areas [25,27,28,30,44]. These observations indicate that while increasing the HGM content up to a certain level improves the mechanical reinforcement and rigidity of the composites, excessive HGM content can lead to a softened composite and higher energy dissipation.

### 3.5. Compressive Test Results of WHGM/PDMS Composites

The compressive properties of PDMS composites filled with WHGMs were evaluated to understand the effect of the WHGM content on mechanical performance. Due to processability limitations, we focused on evaluating the mechanical strength and processability of the PDMS/WHGM composite up to 50 wt.%. Therefore, we investigated mechanical strength and processability in the range of 10 wt.% to 50 wt.%. As shown in Figure 8a, at 10 wt.% WHGM, the composites exhibit a compressive strain of 46.5% at 0.6 MPa, reflecting higher compressibility. Increasing WHGM content to 15 wt.% and 50 wt.%, the strain reaches 16.6% and progressively enhances stiffness and reduces deformation. The results at 0.1 MPa and 0.2 MPa exhibit similar trends, showing reduced maximum strain (Figure 8b–d). This trend is also reflected in the elastic modulus, which increases from 1.28 MPa at 10 wt.% WHGM to 3.59 MPa at 50 wt.% WHGM. The results show a clear trend of decreasing compressive strain with increasing WHGM content, highlighting the role of WHGMs in reinforcing the PDMS matrix. These results illustrate that the WHGM effectively enhances stiffness and compressive strength. The observed behavior is attributed to the uniform distribution and reinforcing effect of WHGM particles within the matrix, which improve the load-bearing capacity and mechanical stability of the composites. These results underscore the potential of the WHGM as an effective filler for tailoring the stiffness and compressive strength of PDMS composites.

### 3.6. Compressive Test Results of B/HGM/PDMS Composites

The incorporation of heavy fillers, like B, significantly increases the density of composites, which can pose challenges in applications, such as aerospace and electronics, where lightweight materials are often critical. To address this, light HGM fillers were introduced to reduce the density of the B/PDMS composites. As shown in Table 1, increasing the HGM content from 5 to 20 wt.% in composites such as B65/HGM5, B50/HGM10, and B45/HGM20 resulted in a substantial density reduction from 1.38 to 0.87 g/cm^3^, confirming the effectiveness of HGMs in lowering the composite density. The compressive properties of these composites were also evaluated to understand the combined effects of B and HGM fillers on mechanical performance. For B65/HGM5, Figure 9a shows a moderate compressive strain of 23.1% at 0.6 MPa, indicating a balance between rigidity and compressibility. Increasing the HGM content to 10 wt.% (B50/HGM10) reduced the compressive strain, reflecting enhanced rigidity and compressive strength. The B45/HGM20 composition, with the highest HGM content, exhibited the lowest compressive strain of 5.9%, indicating the greatest rigidity and compressive strength among the tested samples. The results at 0.1 MPa and 0.2 MPa exhibit similar trends, showing reduced maximum strain (Figure 9b,c). This synergy between B and HGMs not only reinforces the mechanical properties but also significantly reduces the composite’s density, making it more suitable for lightweight applications that demand high compressive performance.

## 4. Conclusions

This study examined the compressive behavior of high-MW PDMS composites incorporating various fillers, including boron (B), hollow glass microballoons (HGMs), tungsten-coated hollow glass microballoons (WHGMs), and a B/HGM combination. The results demonstrate that the PDMS-based matrix accommodates high filler loading, and integrating these fillers strategically can significantly enhance the mechanical performance of the composites. B increased density while improving stiffness and compressive strength. The HGM reduced density, improved mechanical strength, and enhanced energy absorption, making the composites ideal for impact-resistant applications. The WHGM provided an optimal balance of reduced density, increased stiffness, and improved mechanical strength. Notably, the combination of B and HGMs achieved superior mechanical properties while substantially lowering the composite’s density, rendering it suitable for lightweight applications requiring high compressive performance. This study primarily focused on the development and characterization of PDMS composites, without conducting application-specific evaluations. Future research should explore if the functionalities of those composites such as radiation shielding, impact resistance, and lightweight structural integrity could further expand the scope of PDMS composites in advanced applications in aerospace, biomedical, and structural engineering fields. These findings underscore the potential of high-MW PDMS composites to integrate distinct filler characteristics effectively, offering enhanced mechanical properties for use in flexible electronics, medical devices, lightweight structural components, and energy-absorbing systems. This work paves the way for further exploration of multifunctional composites tailored to diverse industrial needs.

## Figures and Tables

**Table 1:** Characteristics of PDMS composites containing B, HGM, and WHGM fillers.

Composition (wt.%)	Hardness (Shore A)	Density (g/cm^3^)		PDMS-Based Polymer Matrix (vol.%)	Filler (vol.%)	Void (vol.%)	Elastic Modulus (MPa)
		Theore.	Measured				
PDMS-based polymer matrix (100)	5.1	1.05	1.05	100	-	-	
B 65	54.8	1.62	1.63	54.1	45.9	0.00	1.86
B 70	60.6	1.69	1.64	46.9	49.9	3.23	1.91
B 75	69.2	1.77	1.72	41.0	56.1	2.96	1.97
B 80	81.5	1.86	1.81	34.5	63.0	2.57	2.01
B 85	75.3	1.95	1.83	26.1	67.6	6.23	2.48
HGM 10	37.8	0.85	0.85	73.3	26.7	0.00	1.95
HGM 20	43.4	0.72	0.70	53.3	43.8	2.92	2.07
HGM 30	56.5	0.62	0.61	40.7	57.2	2.15	2.62
HGM 35	59.9	0.58	0.59	36.1	63.9	0.00	2.99
HGM 40	45.0	0.55	0.55	31.4	68.6	0.00	1.31
WHGM 10	11.3	1.03	1.00	85.7	11.5	2.79	1.28
WHGM 15	13.9	1.02	0.98	79.3	16.9	3.77	1.43
WHGM 30	32.2	0.99	0.96	64.0	33.1	2.90	3.14
WHGM 40	42.0	0.97	0.97	55.4	44.6	0.00	3.19
WHGM 50	48.5	0.95	0.94	44.8	54.0	1.22	3.59
B 65/HGM 5	53.0	1.38	1.35	38.6	59.2	2.18	2.58
B 50/HGM 10	54.1	1.10	1.12	41.8	58.2	0.00	2.75
B 45/HGM 20	51.4	0.87	0.86	28.7	70.6	0.76	10.15

Note: The PDMS-based polymer matrix consists of a PDMS to silica weight ratio of 70:30 (wt.%). “B 65” refers to a mixture containing 65 wt.% of boron and 35 wt.% of the PDMS-based polymer matrix.

## Data Availability

The original contributions presented in this study are included in the article. Further inquiries can be directed to the corresponding author.

## Supplementary Materials

The following supporting information can be downloaded at https://www.mdpi.com/article/10.3390/polym17040500/s1, Figure S1: Surface roughness measurement of PDMS composites: (a) PDMS-based polymer matrix, (b) B 80/PDMS, (c) HGM 40/PDMS, and (d) WHGM 50/PDMS; Figure S2: DSC measurement of PDMS composites: (a) PDMS-based polymer matrix, (b) B 80/PDMS, (c) HGM40 /PDMS, and (d) WHGM 50/PDMS; Figure S3: Cyclic compressive behavior of B/PDMS composites with varying B content: 65, 70, 75, 80, and 85 wt.% under different maximum stresses of 0.1, 0.2, 0.6 MPa; Figure S4: Cyclic compressive behavior of HGM/PDMS composites with varying HGM content: 10, 20, 30, 35, and 40 wt.% under different maximum stresses of 0.1, 0.2, 0.6 MPa; Figure S5: Cyclic compressive behavior of WHGM/PDMS composites with varying WHGM content: 10, 15, 30, 40, and 50 wt.% under different maximum stresses of 0.1, 0.2, 0.6 MPa; Figure S6: Cyclic compressive behavior of B/HGM/PDMS composites with varying B/HGM content: 45/20, 50/10, and 65/5 wt.%. under different maximum stresses of 0.1, 0.2, 0.6 MPa; Figure S7: Cross-sections of B/PDMS composite containing 65, 70, 75, 80, 85 wt.% of B; Figure S8: Cross-sections of HGM/PDMS composite containing 10, 20, 30, 35, 40 wt.% of HGM; Figure S9: Cross-sections of WHGM/PDMS composite containing 10, 15, 30, 40, 50 wt.% of WHGM; Figure S10: Cross-sections of B/HGM/PDMS composite containing 65/5, 50/10, 45/20 wt.% of B/HGM.
