# Supplementary material for: Compression Response of Silicone-Based Composites with Integrated Multifunctional Fillers
Source: Polymers (Basel). 2025 Feb 14;17(4):500. doi: 10.3390/polym17040500 (PMC11859030; doi:10.3390/polym17040500)

polymers

MDPI

Article

Compression Response of Silicone-Based Composites with Integrated Multifunctional Fillers

Ingyu Bak [1], Jihyeon Kim [1], Andrew Jacob Ruba [1], David John Ross [2] and Kwan-Soo Lee [1,*]

[1] MPA-11: Materials Synthesis & Integrated Devices, Los Alamos National Laboratory, Los Alamos, NM 87545, USA; igbak@lanl.gov (I.B.); jhkim@lanl.gov (J.K.); ajruba@lanl.gov (A.J.R.)
[2] MST-7: Engineered Materials, Los Alamos National Laboratory, Los Alamos, NM 87545, USA; dross@lanl.gov
* Correspondence: kslee@lanl.gov

Contents

1. Figure S1. Surface roughness measurement of PDMS composites: (a) PDMS-based polymer matrix, (b) B 80/PDMS, (c) HGM 40/PDMS, and (d) WHGM 50/PDMS. 2

2. Figure S2. DSC measurement of PDMS composites: (a) PDMS-based polymer matrix, (b) B 80/PDMS, (c) HGM40 /PDMS, and (d) WHGM 50/PDMS. 3

3. Figure S3. Cyclic compressive behavior of B/PDMS composites with varying B content: 65, 70, 75, 80, and 85 wt.% under different maximum stresses of 0.1, 0.2, 0.6 MPa. 4

4. Figure S4. Cyclic compressive behavior of HGM/PDMS composites with varying HGM content: 10, 20, 30, 35, and 40 wt.% under different maximum stresses of 0.1, 0.2, 0.6 MPa. 5

5. Figure S5. Cyclic compressive behavior of WHGM/PDMS composites with varying WHGM content: 10, 15, 30, 40, and 50 wt.% under different maximum stresses of 0.1, 0.2, 0.6 MPa. 6

6. Figure S6. Cyclic compressive behavior of B/HGM/PDMS composites with varying B/HGM content: 45/20, 50/10, and 65/5 wt.%. under different maximum stresses of 0.1, 0.2, 0.6 MPa. 7

7. Figure S7. Cross-sections of B/PDMS composite containing 65, 70, 75, 80, 85 wt.% of B. 8

8. Figure S8. Cross-sections of HGM/PDMS composite containing 10, 20, 30, 35, 40 wt.% of HGM. 9

9. Figure S9. Cross-sections of WHGM/PDMS composite containing 10, 15, 30, 40, 50 wt.% of WHGM. 10

10. Figure S10. Cross-sections of B/HGM/PDMS composite containing 65/5, 50/10, 45/20 wt.% of B/HGM. 11

1. Figure S1. Surface roughness measurement of PDMS composites using the Keyence VHX-600 microscope: (a) PDMS-based polymer matrix, (b) B 80/PDMS, (c) HGM 40/PDMS, and (d) WHGM 50/PDMS.

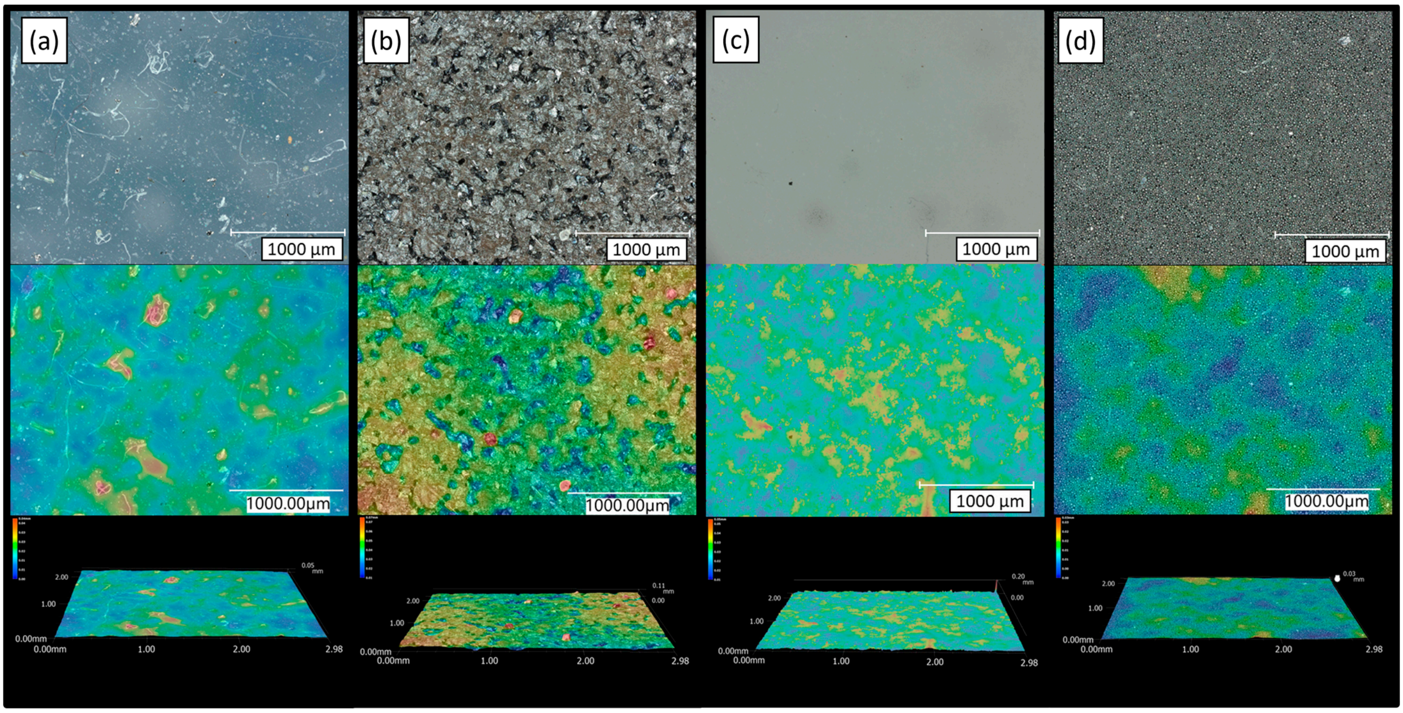

2. Figure S2. DSC measurement of PDMS composites: (a) PDMS-based polymer matrix, (b) B 80/PDMS, (c) HGM40 /PDMS, and (d) WHGM 50/PDMS.

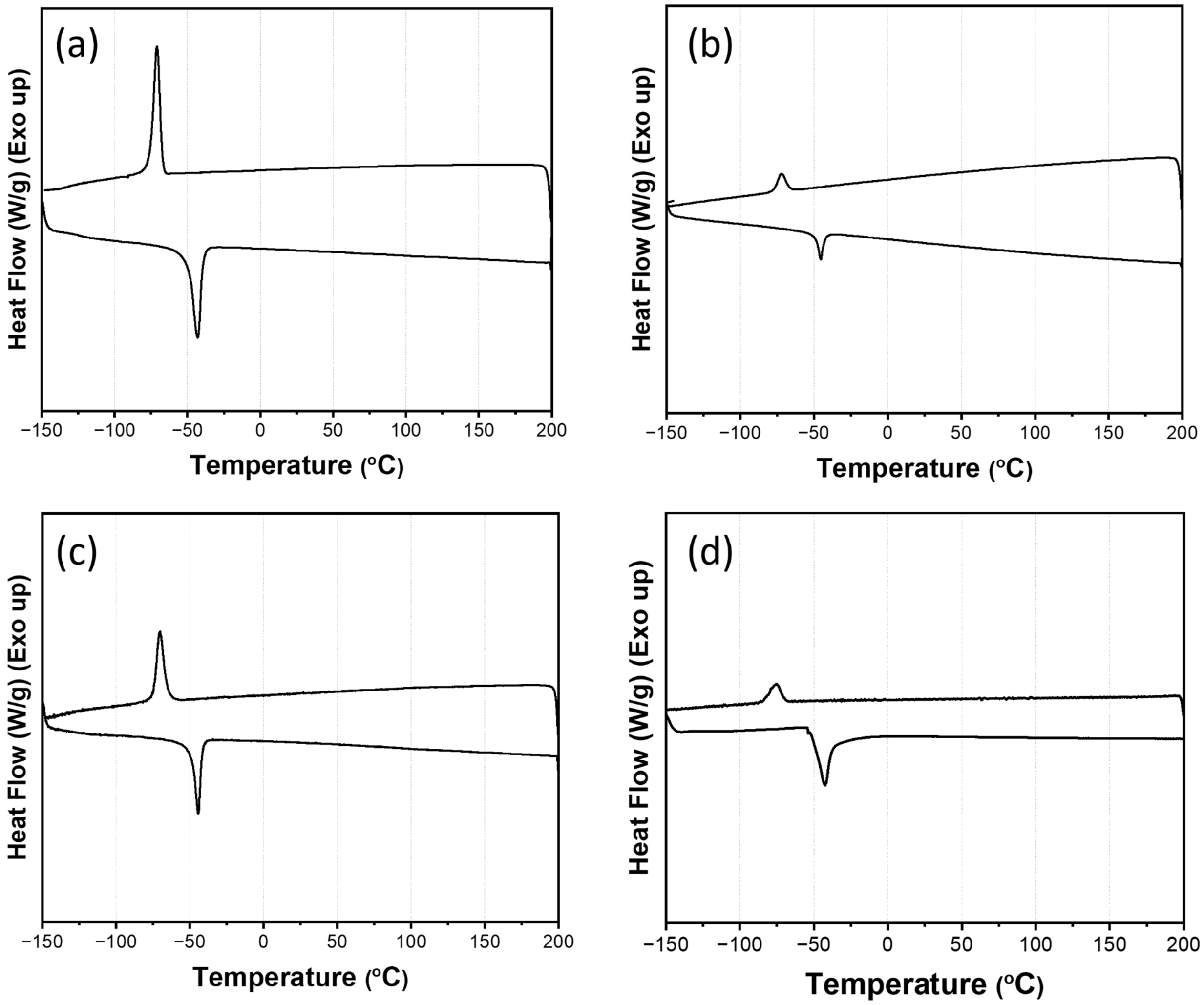

3. Figure S3. Cyclic compressive behavior of B/PDMS composites with varying B content: 65, 70, 75, 80, and 85 wt.% under different maximum stresses of 0.1, 0.2, 0.6 MPa.

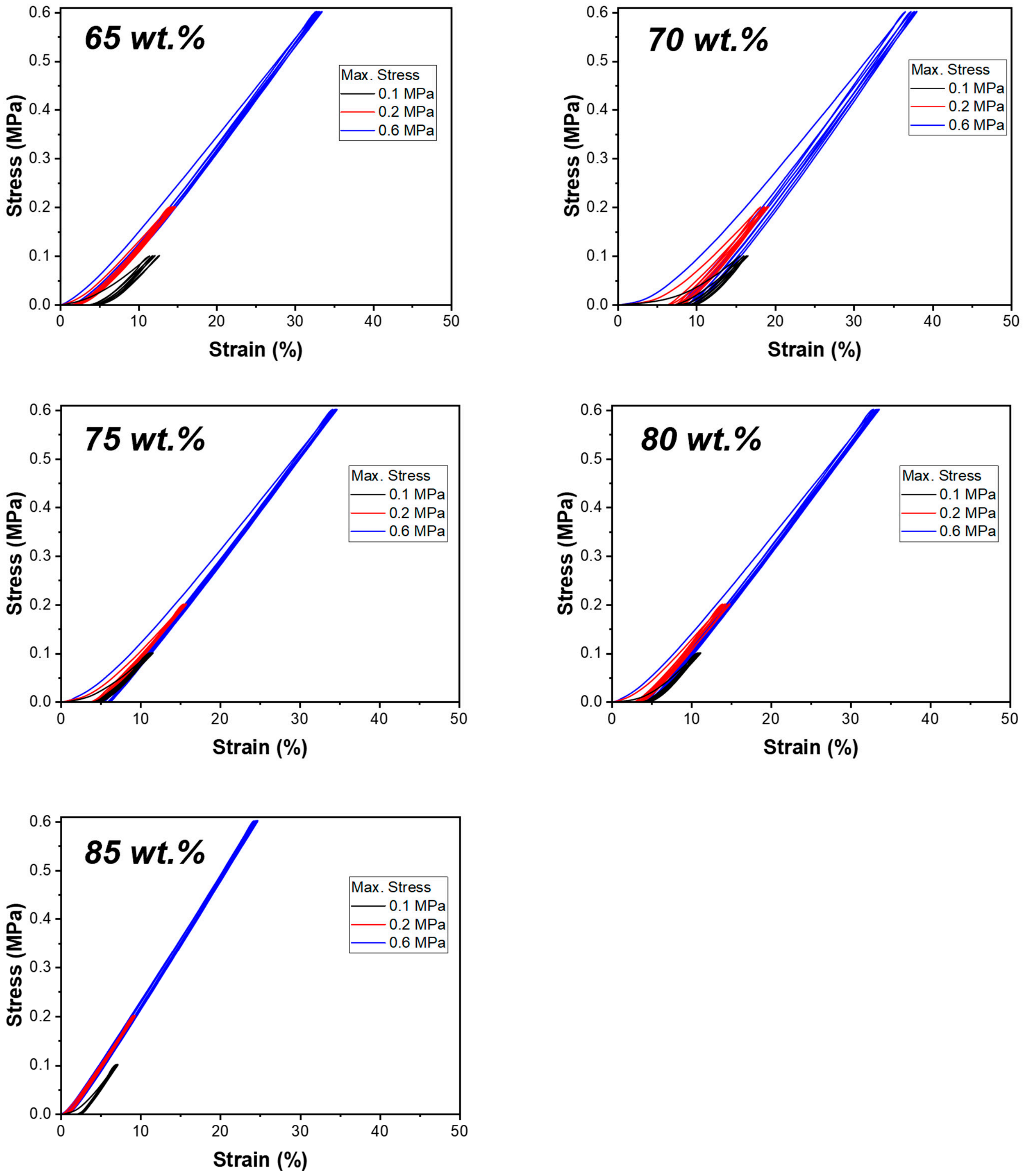

4. Figure S4. Cyclic compressive behavior of HGM/PDMS composites with varying HGM content: 10, 20, 30, 35, and 40 wt.% under different maximum stresses of 0.1, 0.2, 0.6 MPa.

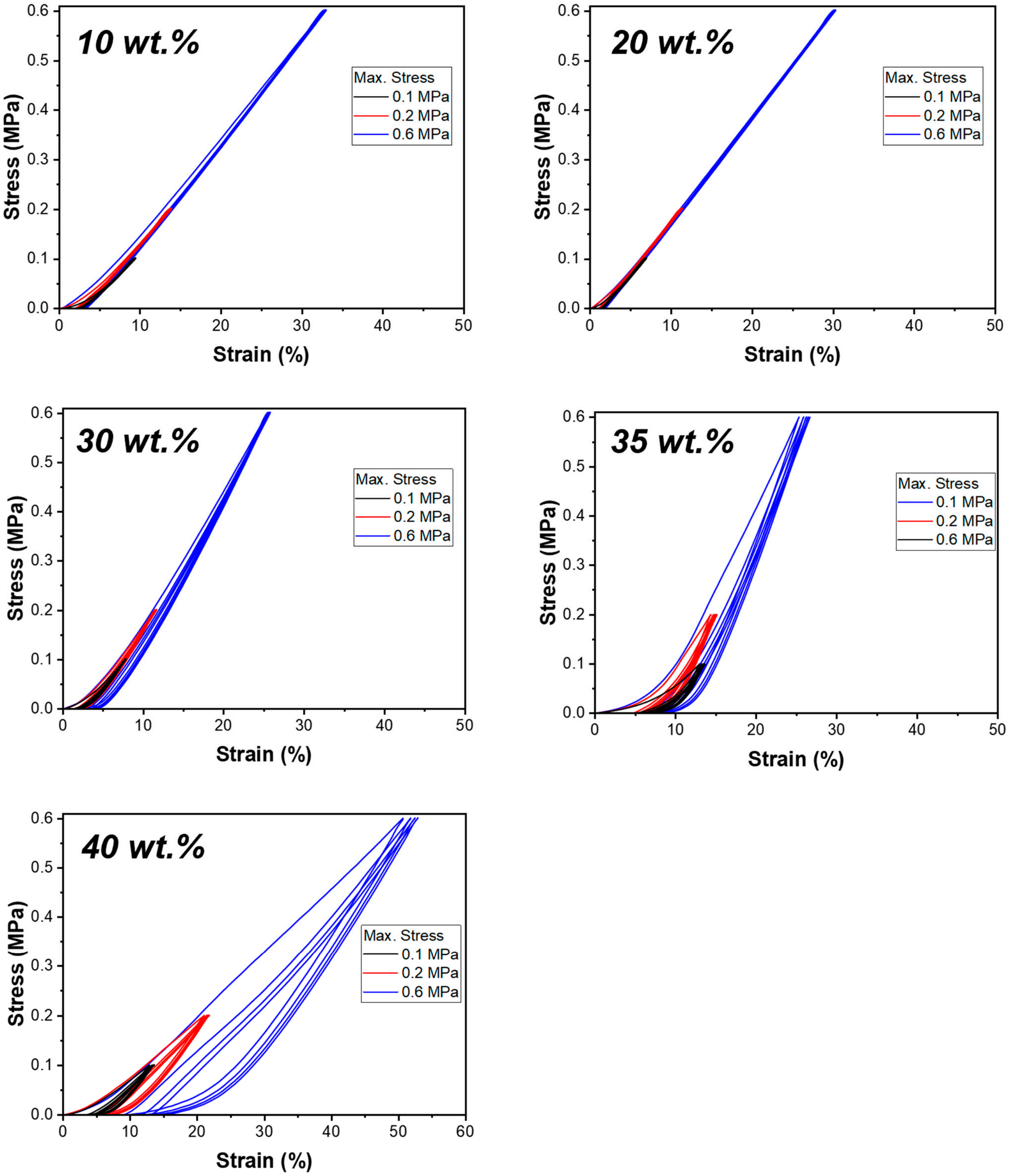

5. Figure S5. Cyclic compressive behavior of WHGM/PDMS composites with varying WHGM content: 10, 15, 30, 40, and 50 wt.% under different maximum stresses of 0.1, 0.2, 0.6 MPa.

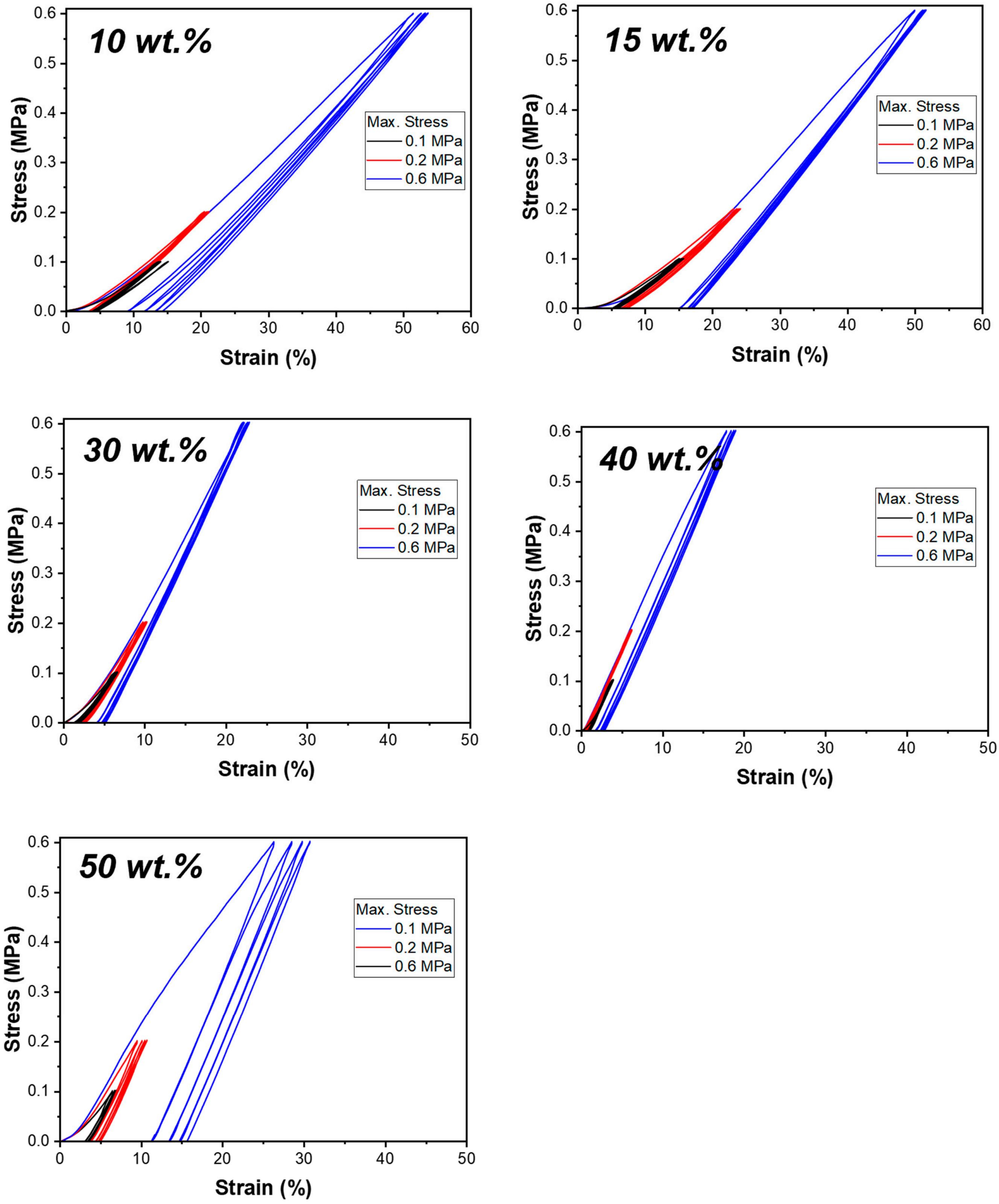

6. Figure S6. Cyclic compressive behavior of B/HGM/PDMS composites with varying B/HGM content: 45/20, 50/10, and 65/5 wt.%. under different maximum stresses of 0.1, 0.2, 0.6 MPa.

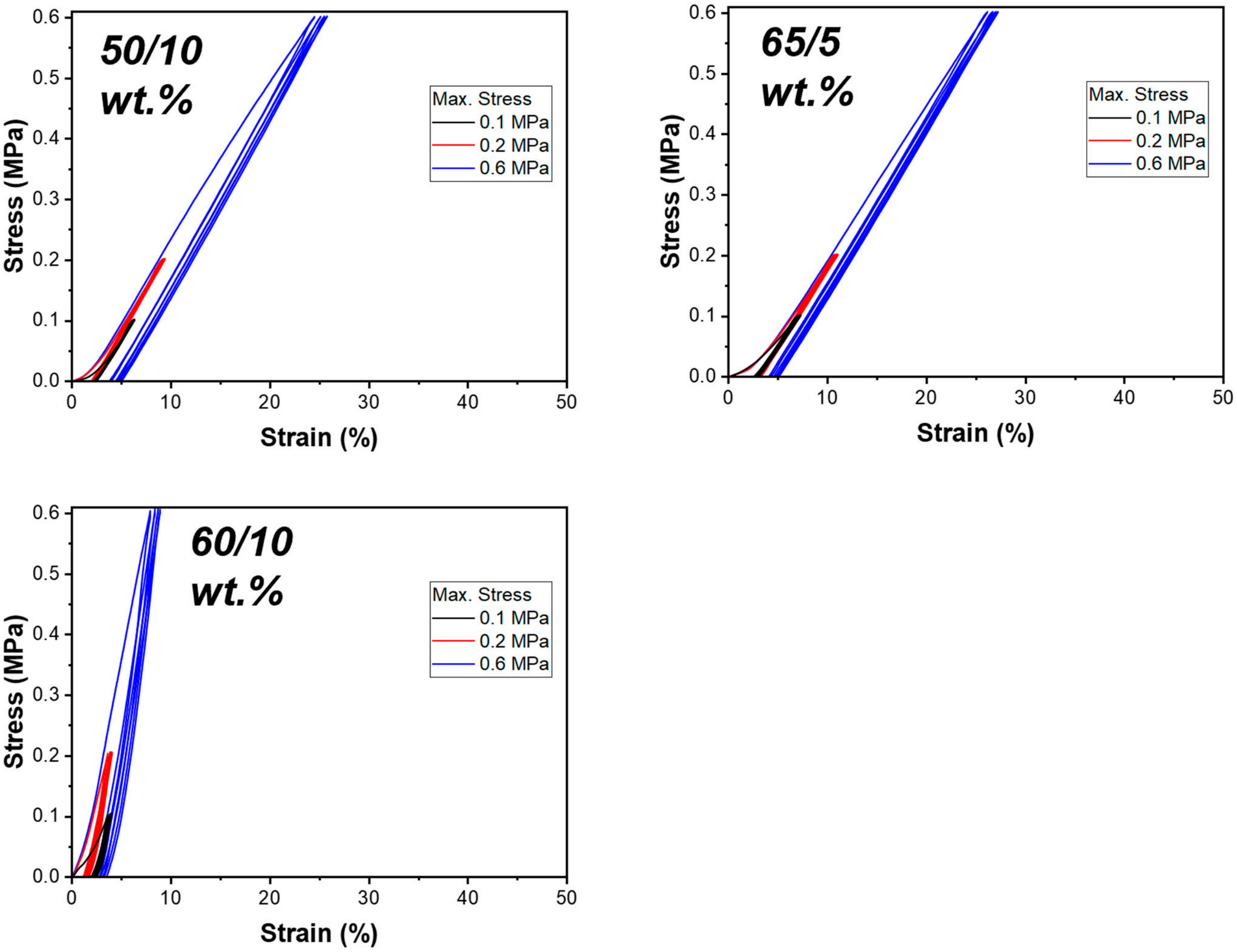

7. Figure S7. Cross-sections of B/PDMS composite containing 65, 70, 75, 80, 85 wt.% of B.

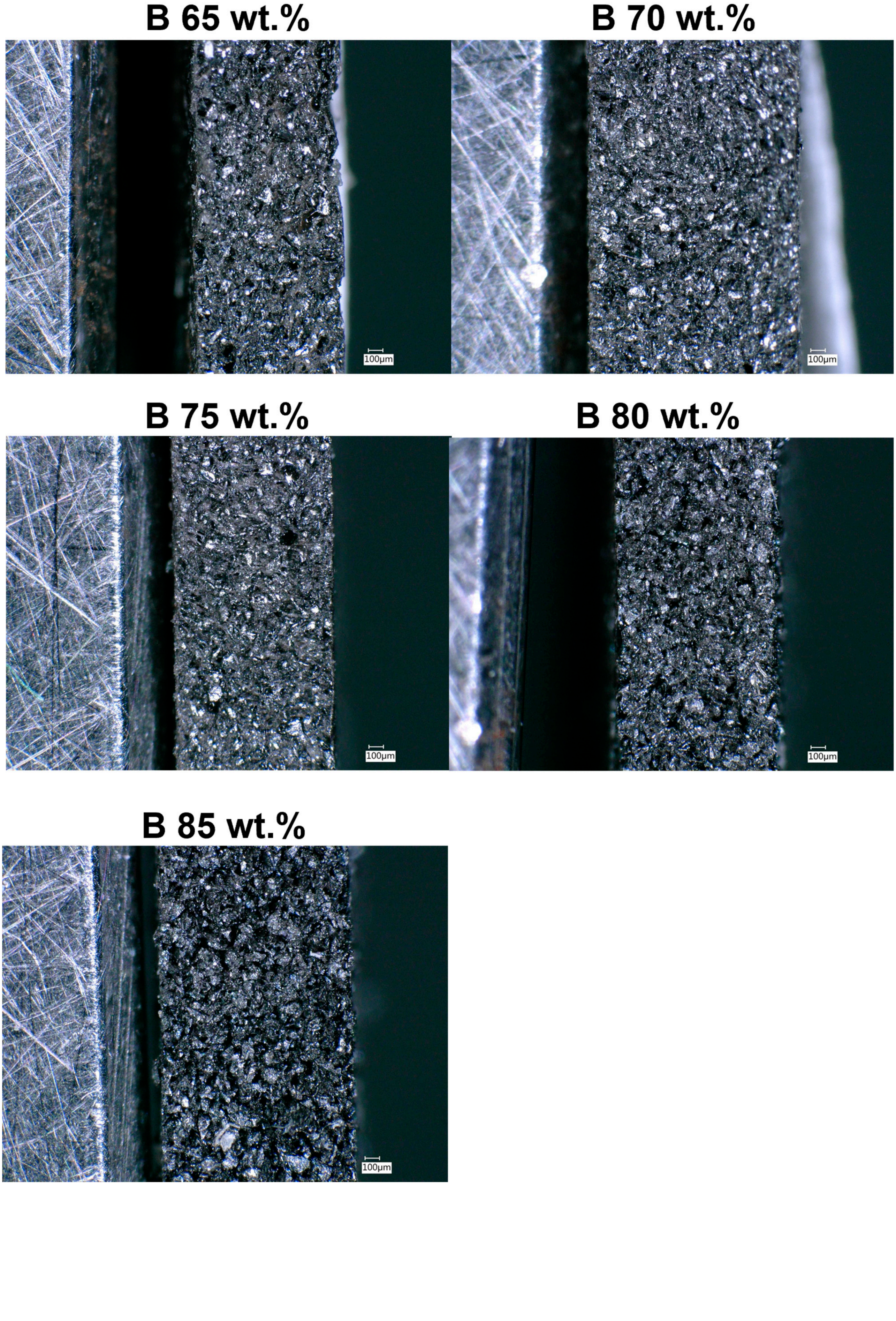

8. Figure S8. Cross-sections of HGM/PDMS composite containing 10, 20, 30, 35, 40 wt.% of HGM.

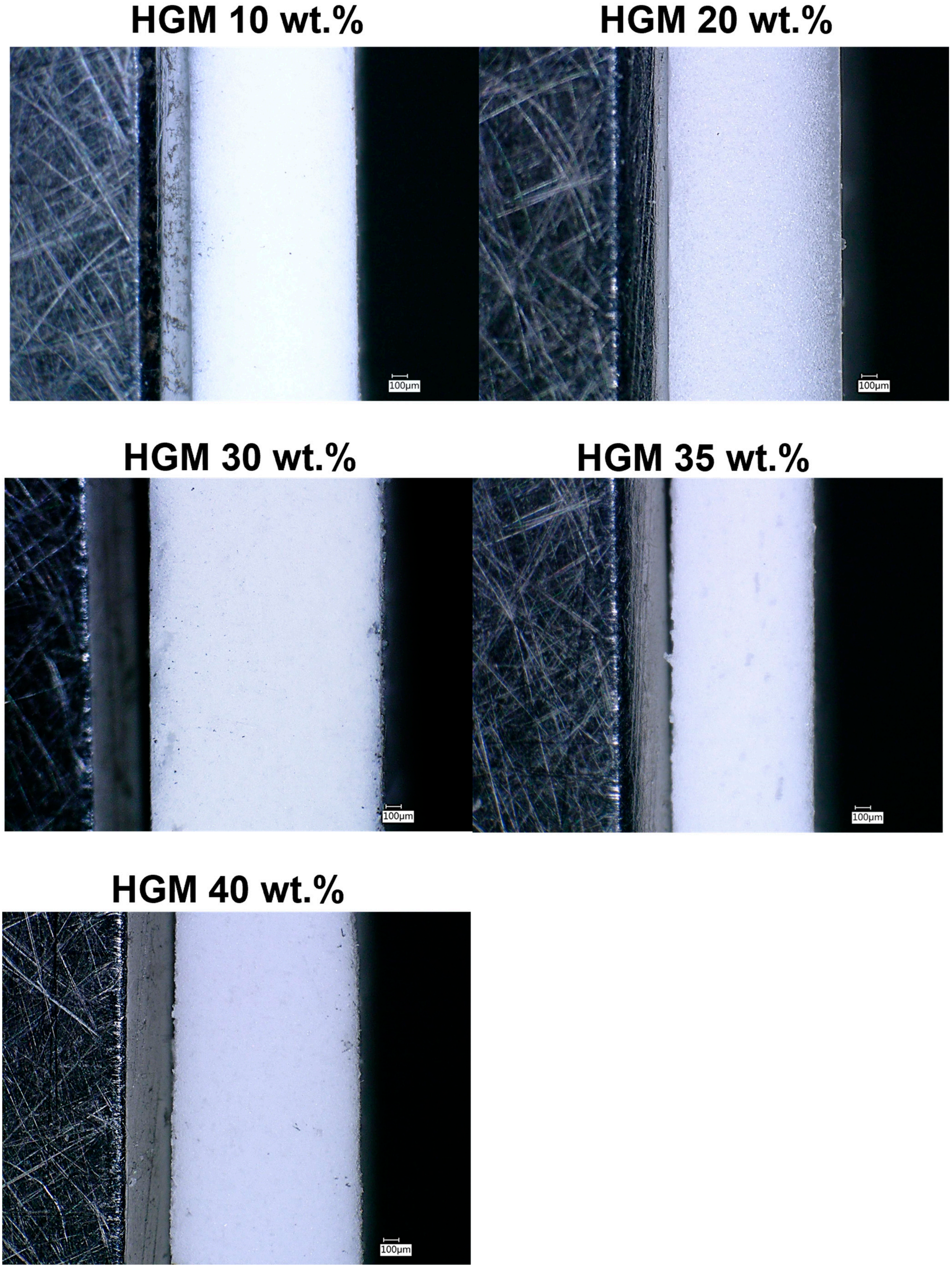

9. Figure S9. Cross-sections of WHGM/PDMS composite containing 10, 15, 30, 40, 50 wt.% of WHGM.

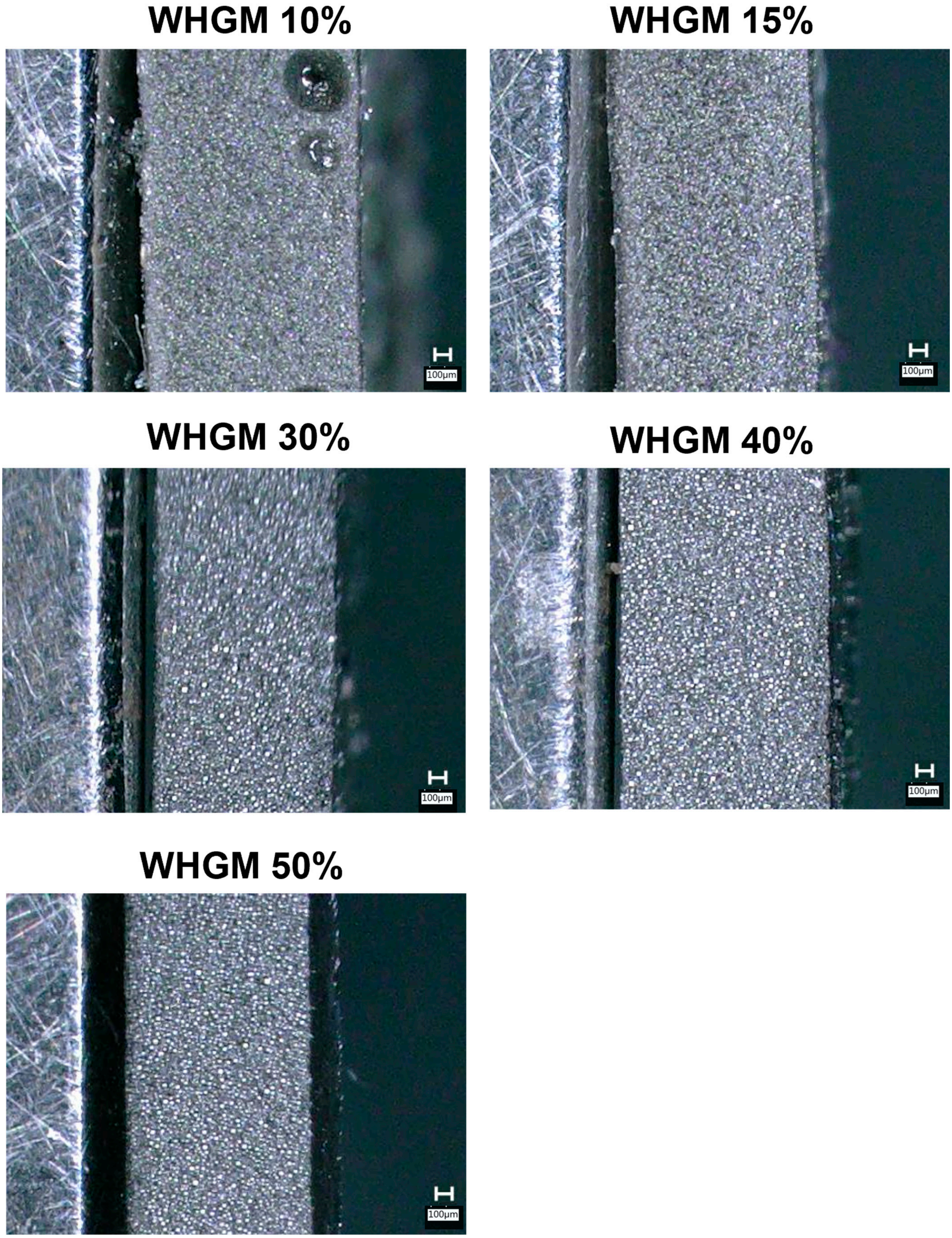

10. Figure S10. Cross-sections of B/HGM/PDMS composite containing 65/5, 50/10, 45/20 wt.% of B/HGM.

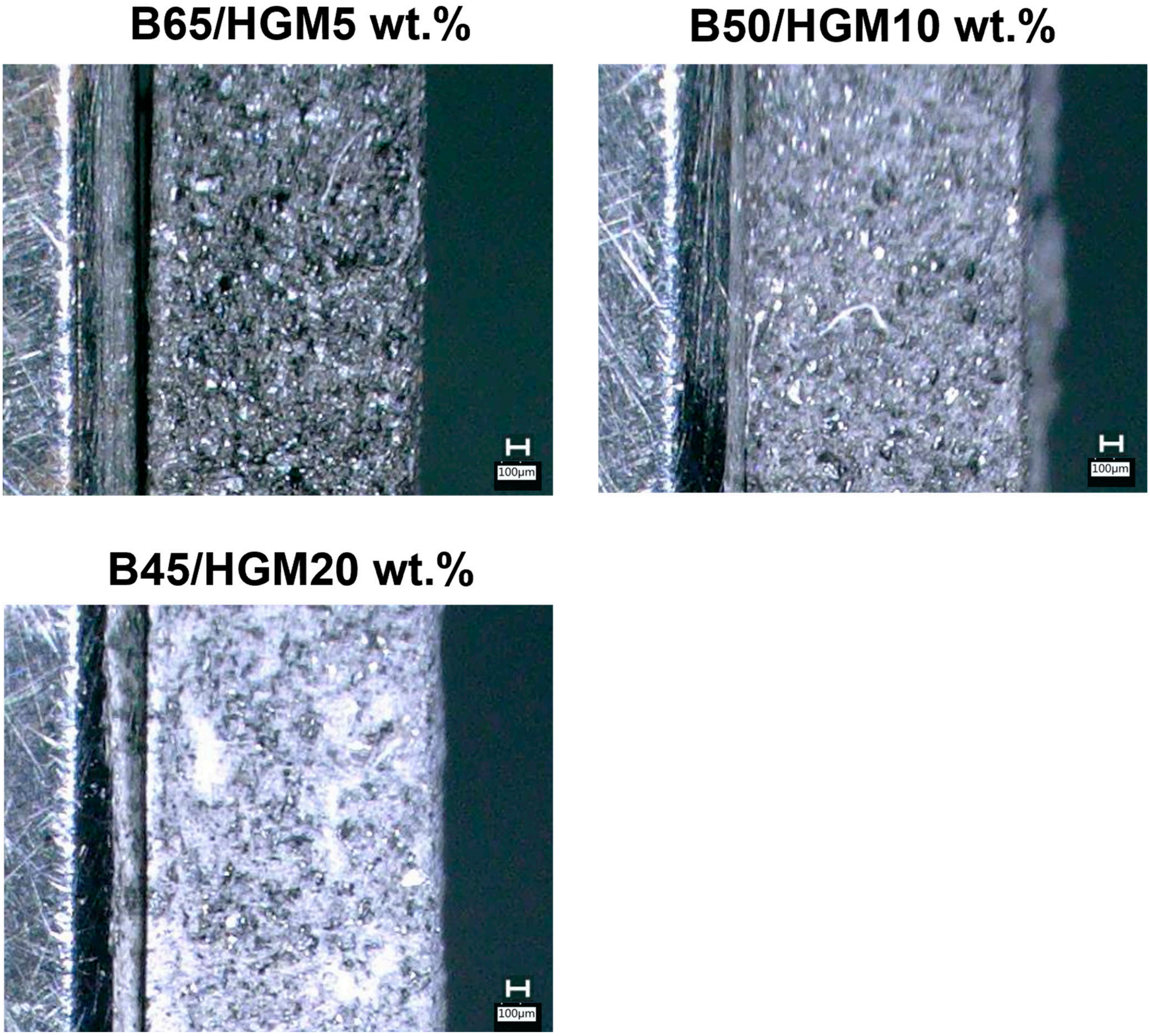

Supplement: Supplementary file 1 [file polymers-17-00500-s001.zip › polymers-3460261-Supplementary Materials.pdf]
